# Analysis of Antiviral Response in Human Epithelial Cells Infected with Hepatitis E Virus

**DOI:** 10.1371/journal.pone.0063793

**Published:** 2013-05-09

**Authors:** Pradip B. Devhare, Subhashis N. Chatterjee, Vidya A. Arankalle, Kavita S. Lole

**Affiliations:** Hepatitis Division, National Institute of Virology, Microbial Containment Complex, Pashan, Pune, India; University of Cincinnati College of Medicine, United States of America

## Abstract

Hepatitis E virus (HEV) is a major cause of enterically transmitted acute hepatitis in developing nations and occurs in sporadic and epidemic forms. The disease may become severe with high mortality (20%) among pregnant women. Due to lack of efficient cell culture system and small animal model, early molecular events of HEV infection are not yet known. In the present study, human lung epithelial cells, A549, were infected with HEV to monitor expression levels of genes/proteins in antiviral pathways. Both live and UV inactivated virus elicited robust induction of inflammatory cytokines/chemokines such as IL-6, IL-8, TNF-α, and RANTES within 12 h of infection. Cells exposed to soluble capsid protein showed no induction suggesting the capsid structure and not the protein being detected as the pathogen pattern by cells. A delayed up-regulation of type I interferon genes only by the live virus at 48 h post HEV infection indicated the need of virus replication. However, absence of secreted interferons till 96 h suggested possible involvement of post-transcriptional regulation of type I IFN expression. HEV infected cells showed activation of both NF-κB and IRF3 transcription factors when seen at protein levels; however, reporter gene assays showed predominant expression via NF-κB promoter as compared to IRF3 promoter. Knockdown experiments done using siRNAs showed involvement of MyD88 and TRIF adaptors in generating antiviral response thus indicating role of TLR2, TLR4 and TLR3 in sensing viral molecules. MAVS knockdown surprisingly enhanced only proinflammatory cytokines and not type I IFNs. This suggested that HEV not only down-regulates RIG-I helicase like receptor mediated IFN induction but also employs MAVS in curtailing host inflammatory response. Our findings uncover an early cellular response in HEV infection and associated molecular mechanisms suggesting the potential role of inflammatory response triggered by HEV infection in host immune response and pathogenesis.

## Introduction

Innate immune system represents the first line of defense against invading pathogens in the hosts. Specific structures such as structural components and replication intermediates of the invading pathogens are recognized by pattern recognition receptors (PRRs) in the host cells resulting in production of type I interferons (IFNs) and proinflammatory cytokines/chemokines to eradicate the pathogen from the cells. This also helps in priming the antigen-specific adaptive immunity. Two families of PRRs, Toll-like receptors (TLRs) and retinoic acid-inducible gene-I like receptors (RLRs) act as sensors of viral infections. TLRs sense the pathogen components on the cells surface and endosomal compartments. In contrast, RLRs survey the cytoplasm for the presence of viral double-stranded RNA (a replication intermediate) and 5′-triphosphate group containing single stranded RNA molecules [1]–[6]. Type I IFNs initiate expression of numerous IFN-stimulated genes (ISGs) in an autocrine or paracrine manner to induce antiviral state in the infected and neighboring cells [6]. Viruses employ different strategies to evade innate immune responses in the host cell for productive infection [6]–[7].

Hepatitis E is largely an acute and self-limiting disease caused by enteric transmission of hepatitis E virus (HEV). Severe manifestation of hepatitis E is more common in pregnant women with high mortality rates (20%). Persistent HEV infections have been recently documented in immunosuppressed patients [8].

Hepatitis E virus is a non-enveloped, single stranded, positive sense RNA virus of size 27–34 nm belonging to *Hepevirus* genus of the family *Hepeviridae*. HEV genome is ∼7.2 kb long with short 5′- and 3′- noncoding regions (NCRs), a 5′- methylguanine cap, a 3′- poly (A) stretch and three open reading frames (ORF1, ORF2 and ORF3) [9]. ORF1 encodes for nonstructural polyprotein involved in viral replication while ORF2 encodes for capsid protein containing, three glycosylation sites [10] and immunodominant epitopes. ORF3 encodes cytoskeleton-associated phosphoprotein [11]. ORF2 and ORF3 overlap with each other and are proposed to be translated from a single, bicistronic mRNA. ORF2 and ORF3 proteins interact with many cellular proteins possibly helping establish HEV infection [12].

It is suggested that hepatic damage in hepatitis E patients is immune mediated and not by the direct replication of the virus [8]; however, the exact mechanism of liver damage is not yet known. Analysis of patients with acute hepatitis E has shown altered frequencies of NK cell subtypes suggesting probable involvement of innate immunity [13]. A microarray study of comparative pathogenesis of HEV and HCV in Chimpanzees has shown attenuated expression of innate response genes in HEV infection as compared to HCV [14]. Studies from our laboratory have shown low levels of IFN-α and comparatively higher levels of inflammatory cytokines/chemokines such as IL-6, IL-8 and TNF-α in acute phase hepatitis E patients [15]–[16].

HEV remains a difficult virus to study *in vitro*. There is a recent report on the propagation of genotype 3 and 4 HEV in liver and lung epithelial cells [17]. Expression patterns of genes that initiate signaling pathways leading to the induction of protective cellular genes during initial phases of HEV infection are not yet known. It was recently demonstrated that HEV inhibits IFN-α signaling and manages to replicate in the presence of IFN-α [18]. However, it is not yet known whether HEV is able to alter IFN induction in the host cells. In the present study we infected lung epithelial cells, A549, with HEV and analyzed the induced antiviral response.

## Materials and Methods

### Ethical Statement

National Institute of Virology, Pune, is the nodal organization in India for investigating suspected viral outbreaks. During epidemic investigations, no ethical clearance is required. For hepatitis outbreaks, the investigating team collects blood and stool samples from the cases for serological diagnosis and PCR based virus detection respectively. Since hepatitis E and hepatitis A have faeco-oral route of transmission water samples are also collected from drinking water sources for virus detection. As the stool samples used in the current study were collected during an epidemic it was not necessary to obtain informed consent of the patients.

### Virus stock preparation

HEV RNA positive (genotype 1, GenBank accession no. DQ459342.1) stool sample collected from a confirmed hepatitis E case (anti-HEV IgM positive) was used to prepare 10% stool suspension and centrifuged at 10000 g at 4°C for 10 min. Supernatant was filtered through 0.22 µm filter and stored at −80°C until further use. Virus purification was carried out by sucrose step gradient centrifugation as described previously [19] and HEV RNA copy number was determined using TaqMan real-time PCR assay [20]. LPS contents of the purified virus stock were checked using LAL chromogenic endotoxin quantitation kit (Pierce). UV inactivation of HEV was carried out as described previously [21]. Influenza A virus (Perth H3N2, NIV SF-33041) stock propagated in embryonated chicken eggs (32 haemagglutinin units/ml) was aliquoted and stored at −80°C until further use.

### Cells and virus infection

Cell lines were either obtained from ATCC (USA) or JCRB (Japan). S10-3 cells were a kind gift from Dr. S. Emerson (NIH, USA) [19], [22]. Hepatoma cells (PLC/PRF5, Huh7, and HepG2), subclonal hepatoma cells (S10-3, and HepG2/C3A) and non-hepatoma cells (Caco2, human colon carcinoma and A549, human lung carcinoma) were infected with HEV with 1∶1 ratio of cells: HEV RNA genome equivalents and harvested at different time intervals. Based upon the results obtained from screening of these cell lines all further work was carried out using A549 cells, propagated in F12-K medium (Invitrogen, Life technologies, USA), supplemented with 10% FBS, penicillin (100 U/ml) and streptomycin (100 µg/ml) at 37°C and 5% CO_2_. Day before infection, cells were either seeded in 6 well (2×10^5^ cells/well) or 12 well (1×10^5^ cells/well) culture plates to achieve 50–60% confluency. Cell monolayer was first washed with serum free medium, OptiMEM (Invitrogen, Life technologies) and then infected with the virus, appropriately diluted in the same medium, for 2 h at 34.5°C. After adsorption of the virus, inoculum was removed and cell layer was washed five times and maintained in F12-K medium with 10% FBS. Influenza virus infection was also carried out similarly.

### Detection of HEV replicative intermediate RNA and proteins: Negative strand specific RT-PCR

Total cellular RNA was extracted from cells using Ribopure RNA extraction kit (Ambion, Life technologies, USA) and detection of negative sense RNA (nsRNA) (replicative intermediate) was done as described previously using tagged primer-based reverse-transcription PCR [23].

### Immunofluorescence assay (IFA)

Cells were trypsinized 24 h before the stipulated time point and plated in Lab-Tek chamber slides (Nunc, Thermo scientific). Next day, cells were fixed with acetone for 30 min, and incubated with anti-HEV mAb (generated against partial ORF2 protein, 458–607 amino acids) at RT for 30 min. After three washes with PBS containing 0.1% Tween-20, cells were incubated with Alexa Fluor 488-conjugated goat anti-mouse antibody (Invitrogen) for 30 min at RT, washed and viewed on FLoid™ Cell Imaging Station (Life technologies, CA, USA).

### Detection of pORF2 binding to cells by Immunofluorescence and flow cytometry

For cell imaging studies, A549 cells were grown on LabTek chamber slides for 24 h and the purified recombinant ORF2 protein [24] was added on the cells (10 µg/ml). Binding was carried out for 1 h at room temperature, following which cells were washed extensively with PBS and fixed with 4% formaldehyde. Staining was done as described above for immunofluorescence assay. For flow cytometry analysis, cells were detached from culture dishes and pelleted at 400×g for 5 min. Cell pellets (1×10^6^ cells) were resuspended in PBS containing 1% BSA (wash buffer). Cells were incubated with 10 µg/ml of pORF2 for 1 h at RT, washed and stained to detect ORF2 protein bound to the cell surface as described above. Control cells were also stained with primary and secondary antibodies without prior pORF2 binding. Acquisition was done on FACS Aria flow cytometer (BD Biosciences). Ten thousand events per sample were collected and retrieved data was analyzed using the FACSDiva software (v.5.2.2, BD Biosciences).

### Stimulation of the cells with poly (I∶C)

Cells were transfected with poly (I∶C)/LyoVec complex (poly I∶C with transfection reagent) (InvivoGen) as per the manufacturer's instructions.

### Gene Expression profiling by TaqMan Low Density Array (TLDA)

Antiviral genes (n = 95) and 18 s rRNA as endogenous control were chosen for the study and the array cards were procured from Applied Biosystems (USA) (Table S1). Total cellular RNA extracted using Ribopure RNA extraction kit (Ambion) was checked for integrity and quantified (ND-1000, Nanodrop Technologies). cDNA was prepared using High-Capacity cDNA Archive kit (Applied Biosystems, USA). After ensuring efficient cDNA synthesis, 125 ng (RNA equivalent) of the cDNA was loaded in each port of the TLDA card and run on 7900 HT system (Applied Biosystems). Relative quantification was done using RQ Manager Software.

### Quantitative PCR and ELISAs for interferons, cytokines and chemokines: Quantitative real-time PCR (qRT-PCR)

Verification of the TLDA data was done using individual SYBR green-based quantitative reverse transcription PCR assays for selective genes. The cDNAs prepared using the above method were analyzed on 7300 Real-Time PCR system (Applied Biosystems, USA). GAPDH was used as a housekeeping gene to normalize the RNA input. RNA from mock infected cells was used as the calibrator and relative gene expression analysis was carried out using SDS2.2 software (Applied Biosystems, USA). Primers used for the quantitative analysis are enlisted in (Table S2).

### ELISAs

Cell culture supernatants from the experimental cells were assessed for the interferon, cytokine and chemokine levels as per the manufacturer's instructions. The kits used were IFN-α/β (PBL, Piscataway, NJ), IL-6 (Detection limit 10–2000 pg/ml), RANTES (31.2–2000 pg/ml) (Invitrogen), TNF-α (2–250 pg/ml) (IMGENEX) and IL-8 (31.2–2000 pg/ml) (R & D Systems, USA).

### Luciferase assay

A549 cells were seeded into 12-well plates and co-transfected 24 h later with 500 ng of pNF-κB -Luc (firefly luciferase) (Stratagene) or pIFN-β-Luc and 5 ng of pGL4.75 hRluc/CMV (Renilla luciferase) (Promega) plasmid DNA by using Xfect transfection reagent (Clontech). After 24 h cells were infected with either HEV or UV inactivated HEV (HEV-UV), influenza A virus or left without infection. Cell lysates were assayed using Dual Reporter Assay kit (Promega) on Perkin Elmer 2030 Reader (Victor ×3). Firefly luciferase values were normalized with Renilla luciferase values to normalize transfection efficiencies. Luciferase values obtained from the uninfected cells were subtracted from experimental samples to normalize DNA mediated background expression.

### Type I IFN sensor assay

Stimulation of HEK-Blue™IFN-α/β cells (InvivoGen, San Diego, CA) cells with human IFN-α/β activates JAK/STAT/ISGF3 pathway to induce secreted alkaline phosphatase (SEAP) which can be quantitated with QUANTI-Blue reagent (InvivoGen, San Diego, CA). Virus infected cell culture supernatants were used to stimulate HEK Blue cells for 18–20 h and assayed as per the manufacturer's instructions. A standard curve generated with human recombinant IFN-α (Sigma Aldrich, USA) was used to determine IFN levels in the cell supernatants.

### siRNA assays

The siRNAs targeting human TICAM-1/TRIF (148022), MyD88 (4615) and non-targeting control were obtained from Dharmacon (Thermo Fisher Scientific, USA). Human MAVS siRNA (sc-75755) was obtained from Santa Cruz Biotechnology Inc (USA). A549 cells were transfected either with control (100 nM) or other siRNAs using HiPerFect transfection reagent (Qiagen, Hilden, Germany) and 24 h later infected with the virus. The supernatants were then assayed for cytokine/chemokine levels.

### Immunoblotting

The primary antibodies used were - mAbs mouse NF-kB (p65), anti-nucleolin, anti-IκBα, (Invitrogen), anti-phospho-IkBα (Ser32), anti-MyD88, anti-TRIF polyclonal antibodies, mAb anti-phospho IRF3 (Ser396) (Pierce Biotechnology, Rockford, USA), anti-IRF3, anti-actin(Sigma) and anti-MAVS (ProSci Incorporated, CA, USA). Cells were washed with ice-cold PBS and lysed in buffer (25 mM Tris-HCl, pH 7.5, 137 mM NaCl, 1% NP-40, 2 mM EDTA, 1 mM PMSF). Nuclear and cytoplasmic extracts were prepared using NE-PER kit (Pierce Biotechnology). Equal amounts of protein extracts (20 µg) were analyzed on 10% SDS-PAGE and blots were developed with Amersham ECL Western blotting detection reagents (GE Healthcare, Piscataway, NJ) followed by exposure to X-ray films. For re-probing, membrane was incubated in western blotting stripping buffer (Thermo Scientific) for 15 min at RT and processed for staining.

### Densitometric scanning

Western blot images were scanned on AlphaImager 3400 gel imaging system and densitometric analysis was performed on unsaturated blots by using Alpha Innotech FC software. Relative densitometric value (RDV) for each experimental band was calculated by normalizing the absolute intensity of experimental band with respect to that of the control (actin/nucleolin) bands.

### Statistical analyses

All experiments were performed at least three times. Data is presented as mean ± standard deviation (SD). Statistical analysis was performed on ELISA results using GraphPad Prism software and p-values<0.05 were considered as significant.

## Results

### Selection of cell culture model

HEV RNA copies of the purified viral stock were determined to be 1.4×10^6^ copies/ml. LPS contents of the viral inoculum was 0.8 EU/ml (<1 EU/ml).The stock was diluted appropriately as needed for the infection. While number of cell lines have been shown to support HEV replication, it was essential to use an appropriate model to analyze the interactions between HEV and signaling pathways in the host. HEV does not show cytopathic effect and in absence of robust replication any negligible increase in copy number would not be considered significant by quantitative real time PCR. Under these circumstances, replication can be demonstrated by showing presence of negative strand RNA (replicative intermediate) and newly formed ORF2 protein. To optimize parameters used for monitoring successful virus replication different cells were infected with HEV and analyzed by negative strand RNA detection assay and IFA at different time intervals up to 12 days post infection (p.i.). Considering our previous observations that-i) negative sense RNA is encapsidated along with positive sense genome in hepatitis E virions, ii) negative sense RNA detection could be false positive if reaction contains ≥10^6^ copies of positive sense RNA [23]; we decided to use 10^5^ genome equivalent copies of the virus/well for cell infections in the present study. All the infected cells were processed for negative strand detection at T0 (zero hour time point) and the results were always negative. Hence, infected samples would be positive for negative strand only if virus replicates. All examined cell lines supported HEV replication as indicated by negative sense RNA positivity. HepG2, Caco2, PLC/PRF5, Huh7 and S10-3 cells were positive for negative sense RNA from 96 h post infection while A549 and HepG2/C3A cells were positive from 8 h post infection.

**Figure 1:**
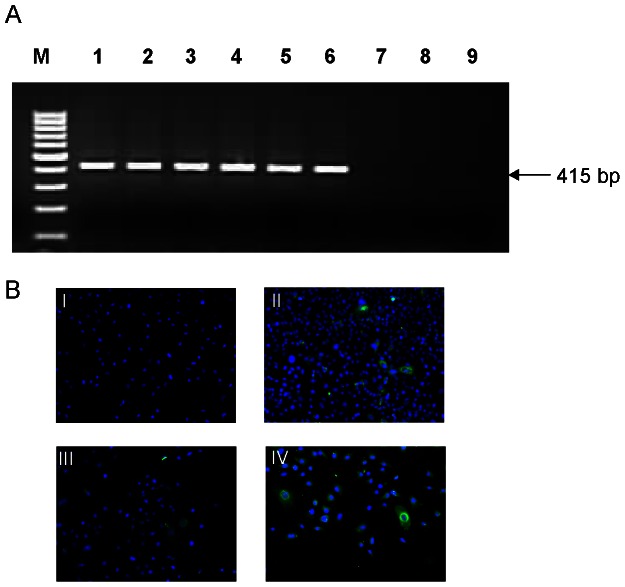
Infection of A549 cells with HEV. (**A**) Negative strand RNA detection: Total RNA isolated from HEV infected A549 cells were processed for negative strand specific tag primer- based reverse transcription PCR. A representative 2% agarose gel of four independent experiments shows PCR product (415 bp) and the lanes are- cells infected with HEV after 8 h (1), 12 h (2), 24 h (3), 48 h (4), 72 h (5), 96 h (6); mock-infected cells (7); cells infected with UV-inactivated HEV after 12 h (8) and 24 h (9), 100 bp DNA ladder (M). (**B**) Immunofluorescence assay: Indirect immunofluorescence microscopy for the detection of HEV ORF2 protein: A549 cells mock infected (I) or infected with HEV were stained with ORF2 specific monoclonal antibodies (green) at 48 h (II), 72 h (III) and 6 days posi-infection (IV). The nuclei (blue) were counterstained with 4′, 6′-diamino-2-phenylindole (DAPI), (Magnification 460×).

All tested cells were positive from 6–8 days post HEV infection when newly synthesized ORF2 protein was detected by immunofluorescence assay (IFA). With previous reports that the transformed hepatoma cells have impaired double stranded RNA and virus-activated IFN responses [25]–[26], HepG2/C3A and A549 cells, which showed negative sense RNA at early time points were stimulated with poly (I∶C) (1–5 µg/ml) to check for their response. A549 cells secreted IFN-β after 8 h (117.5 pg/ml) and levels increased up to 2105 pg/ml at 48 h with 1 µg/ml poly (I∶C). Poly (I∶C) concentrations above 2 µg/ml induced apoptosis within 24 h of exposure. HepG2/C3A cells did not show IFN-β secretion nor cell death with any of the tested poly (I∶C) concentrations (data not shown). Considering the response to poly (I∶C) and permissiveness for HEV infection (Figure 1A, 1B) A549 cells were used for further experiments. At 6 days p.i., these cells showed 15–20% IFA positivity indicating successful infection. UV exposure of the virus for 30 min resulted in complete inactivation as the cells infected with this virus remained negative for both HEV negative sense RNA and IFA up to 12 days.

### Gene expression profile in HEV infection

To monitor the expression levels of different genes in response to virus infection, A549 cells were infected with either HEV or HEV-UV or influenza A virus (known to activate antiviral pathways in A549 cells) [27]–[29]. Results obtained with low density arrays showed differentially expressed genes which are functionally categorized below (Table 1). Expression of selective cytokine/chemokine genes was validated with the SYBR green real-time PCR quantitative assays (Fig. S1).

**Table 1:** Summary of significantly upregulated genes in HEV infected A549 cells analyzed by TaqMan Low Density Array (TLDA).

Pathway/Function	Gene	HEV and (HEV-UV)*					H3N2
		12 h	24 h	48 h	72 h	96 h	12 h
Pattern Recognition receptors (PRRs)	TLR2	4.3 (0.6)	2.7 (1)	10.5 (0.8)	3.1	4.1 (1)	1.1
	TLR3	0.7 (0.2)	0.6 (1)	5 (0.4)	1.4	0.6 (0.2)	7.3
	TLR4	6.9 (0.1)	2.1 (14.5)	40 (0.4)	7.4	13.8 (1.5)	1.3
	DDx58/RIG1	2.1 (0.1)	1.4 (1.2)	4.3 (0.2)	9.1	21.7 (0.8)	13.2
	IFIH1/Mda5	0.8 (0.4)	1.1 (1.6)	5.9 (0.4)	2	1.5 (0.6)	31.3
Proinflammatory cytokines/chemokines	CCL20	69.6 (0.5)	50 (4.6)	4.8 (0.9)	30.7	261 (6.2)	40.4
	IL-6	77.2 (2.2)	28.2 (2.8)	11.2 (1.2)	9.9	175 (4.9)	28
	IL-8	38.9 (4.1)	30.5 (2.9)	50 (4.5)	21.5	96.6 (8.1)	27.5
	TNF alpha	42 (0.8)	9.4 (1.4)	60 (1.6)	174.3	250 (13.1)	116.9
	LTA	2.9 (0.03)	2 (7.7)	1.2 (0.1)	7.8	103 (0.5)	2.6
	TRAIL	2.3 (0.06)	1.8 (2.5)	14.7 (0.3)	3.5	2.2 (0.3)	52.4
	IRAK2	9.8 (0.4)	3.3 (0.9)	3.1 (0.4)	6.5	11.3 (1)	4.8
	TNFAIP3/A20	11.9 (0.6)	6.1 (2.1)	9.1 (0.6)	5.1	50.3 (1.5)	18.5
Interferons	IFNα	2.7 (0.4)	2 (1.5)	3.6 (0.5)	2.3	11.5 (0.6)	2
	IFNβ	5.9 (0.2)	0.7 (0.7)	125.3 (0.1)	221	122 (0.7)	407
	IFNω	5.6 (0.2)	6.2 (0.4)	9.5 (0.1)	13.6	4.7z(0.3)	0.5

Gene expression levels are given as average fold change of three independent experiments. (Fold change ≥2 considered to be up-regulated genes, * RQ values obtained from UV inactivated HEV infection, Gene expression analyzed from Influenza A (H3N2) virus infected A549 cells (12 h) included as positive control).

### Pattern Recognition Receptors (PRRs)

Transcription of cytosolic receptor, RIG-I/DDX58 was initially low (2.1 folds at 12 h) and increased subsequently as virus replication proceeds from 48 to 72 h post HEV infection (4.3–21 folds). There was only a transient increase of MDA-5/IFIH1 expression at 48 h (5.9 folds). Expression of RIG-I and MDA5 remained unchanged with HEV-UV. Cell surface TLRs such as TLR2 and TLR4 were also up-regulated only in live HEV infected cells. Expression of TLR3, which recognizes double stranded RNA (dsRNA) remained unchanged in HEV and HEV-UV infected cells. Influenza virus induced RIG-I (13 folds), MDA-5 (31 folds) and TLR3 (7.3 folds) at 12 h post infection (Table 1). Due to impaired TLR7 and TLR8 genes in A549 cells their levels could not be analyzed [30].

### Inflammatory Cytokine/chemokine Genes

CCL20 was up-regulated at all time points in HEV infected cells. HEV-UV infection also induced CCL20 to lower levels (Table 1). CCL5/RANTES expression levels were significantly higher with both live (2.5–10 folds) and HEV-UV (10–20 folds) at 24 h (Fig. S1). Other significantly up-regulated genes in HEV infected cells were IL-6, IL-8, TNF-α, TNF family member LTA and TNFSF10/TRAIL. Except LTA there was low level up-regulation of these genes also with HEV-UV infection (Table 1).

### Type I Interferons

IFN-α showed a low level of up-regulation from 12 h onwards post HEV infection. Levels of IFN-β were initially low (∼6 folds) but increased significantly at 72 h (221 folds). HEV infected cells showed upregulation of IFN-ω (5–13 folds) during 12–96 h (Table 1). Importantly, IFN induction required live HEV infection as none of the type I IFN genes were up-regulated with HEV-UV. This showed that although HEV managed to keep transcription of type I IFN genes at low levels in the early phases of infection, levels increased significantly after 48 h. In tune with type I IFN gene up-regulation, interferon stimulated genes such as IRF1, IRF7, IRF9, ISG15, MX1, OAS1, GBP1, GBP2, IFIT1, IFIT2 and PKR showed delayed induction at 48 h post HEV infection (Table 1, Table S3).

### Other genes

IRAK 2 (Interleukin-1 receptor-associated kinase 2), known to play crucial role in TLR signaling pathway and activation of NF-κB, was significantly induced only in live HEV infected cells (3–11 folds). TNFAIP3/A20, a negative regulator of NF-κB signaling was also induced only in live HEV infected cells (Table 1).

### Detection of secreted type I interferons

**Figure 2:**
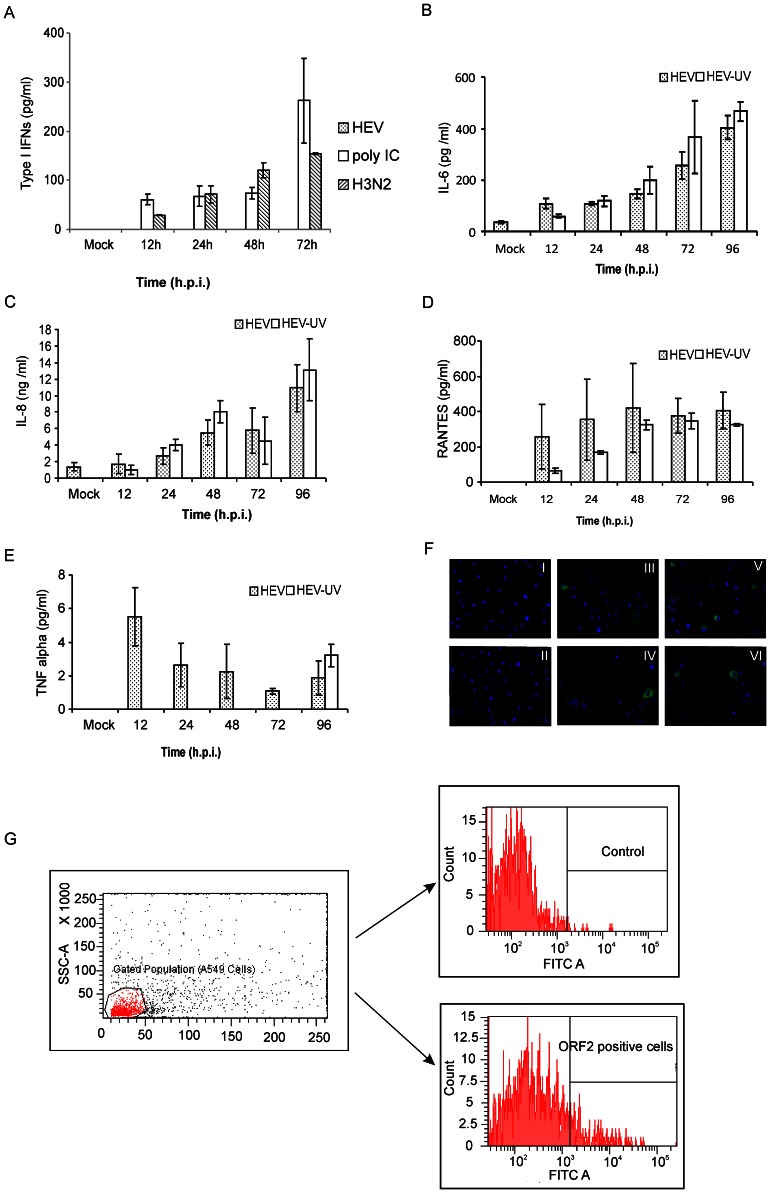
Cytokines/chemokines in the cell supernatants. (**A**) HEK-Blue™ IFNα/β responsive assay: The interferon responsive HEK Blue cells were exposed for 18 h to cell free supernatants from mock infected, HEV/H3N2 infected or poly (I∶C) transfected A549 cells at indicated time points and type I interferon levels were determined from a standard curve generated by similarly exposing the cells with recombinant interferon-α. Results are shown as mean ± SD of three independent experiments. (**B**–**E**) Secreted levels of the proinflammatory cytokines/chemokines: A549 cells were infected with either live or UV inactivated HEV and culture supernatants were tested at indicated time points using ELISA assays- IL-6 (B), IL-8 (C), RANTES (D) and TNF-α (E). Mock represents cell culture supernatant from non-infected cells (12 h time point) processed for respective ELISAs. Data are mean ± SD of four independent experiments (h.p.i; hours post infection). (**F**). Soluble HEV ORF2 protein interacts with the cell surface of A549 cells: A549 cells grown on LabTek chamber slides were incubated with 10 µg/ml of ORF2 protein for 1 h and after extensive washes with PBS, cells were fixed with 4% formaldehyde and stained for the detection of ORF2 protein as described above, (panel I and II) control cells stained with primary and secondary antibodies without addition of ORF2 protein- (panel III-VI) Cells positive for ORF2 protein are green and the nuclei stained with DAPI are blue. (**G**) Flow cytometry analysis of the binding of ORF2 protein to A549 cells: The staining was done as described in the methodology. The histogram shown is representative of two independent experiments. Left panel represents the gated population of A549 cells, right panels are control (without ORF2) and experimental (ORF2 exposed) cells.

There was no measurable secretion of IFN-α and IFN-β until 96 h in culture supernatants of the cells infected with either HEV or HEV-UV (data not shown). Considering the fact that ELISA assay has lower sensitivity (detection range 25 to 2000 pg/ml), another sensitive IFN α/β assay (detection range 12.5 to 500 pg/ml) with HEK Blue cells was used however, the results remained negative. As mRNAs are eventually translated into proteins, detectable levels of IFN-β were expected to be secreted 72 h onwards in the HEV infected cell supernatants. However there was no correlation between the levels of mRNA and protein during IFN synthesis. Supernatants tested from the cells stimulated with poly (I∶C) RNA or infected with influenza A virus showed secretion of IFNs from 24 h onwards (Figure 2A).

### HEV induces proinflammatory cytokines/chemokines

Significantly higher levels of IL-6 (108–400 pg/ml) (Figure 2B), IL-8 (1.7–11 ng/ml) (Figure 2C) and RANTES (258–420 pg/ml) (Figure 2D) were detected from 12–96 h p.i. in cells infected with HEV and HEV-UV. Low level TNF-α secretion (2.5–5.5 pg/ml) was detected from 12–48 h p.i. with HEV and at 96 h with HEV-UV (Figure 2E). Comparable induction of proinflammatory cytokines/chemokines with HEV and HEV-UV prompted us to explore the capability of capsid (ORF2) protein alone in inducing pathways leading to cytokine synthesis. For that, purified recombinant HEV ORF2/capsid protein (glycosylated form, expressed in baculovirus system) [24] was allowed to bind to A549 cells and cells were analysed to detect cell bound ORF2 protein. Flow cytometry and IFA analysis of these cells showed 8–10% positivity (Figure 2F and 2G). Cells incubated with various concentrations (1–5 µg/ml) of ORF2 for 2 h did not induce cytokines/chemokines (data not shown). 12 h onwards, the positive control, influenza A virus showed significantly higher levels of all four cytokines/chemokines (Fig. S2).

### Activation of IRF3 and NF-κB

**Figure 3:**
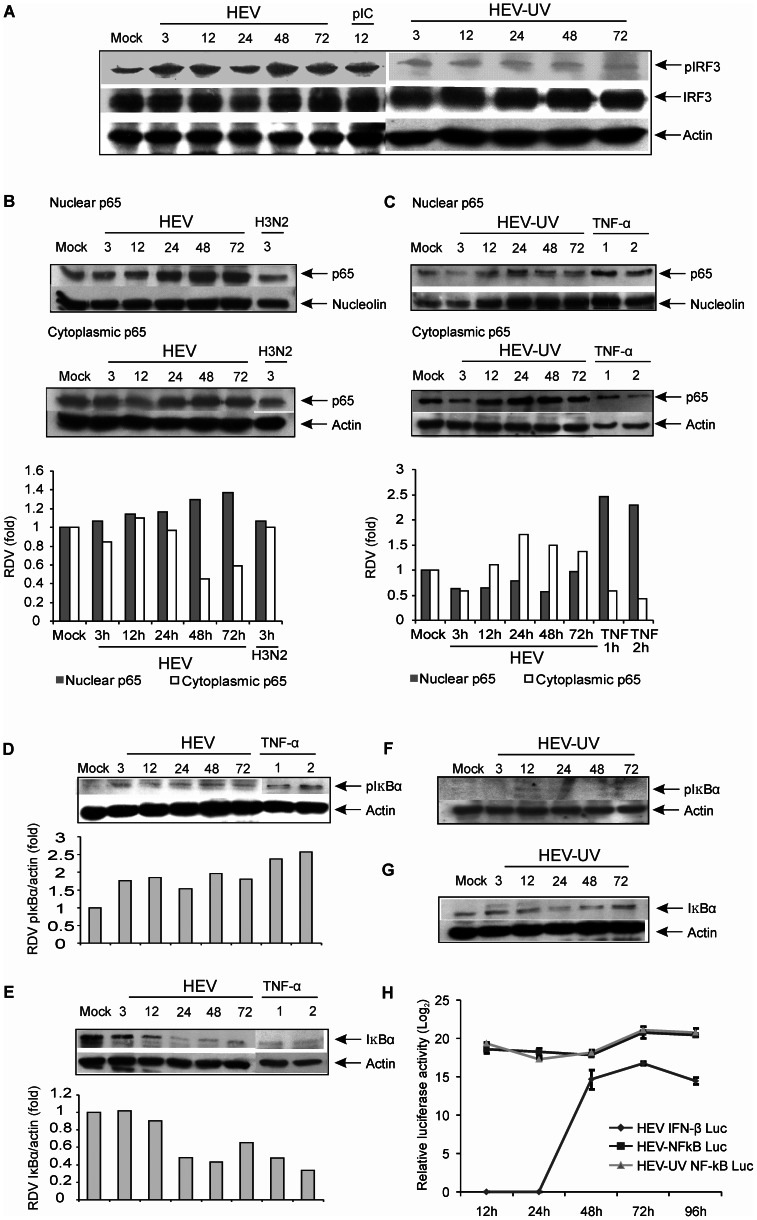
HEV infection induces IRF3 and NF-κB activation in A549 cells. (**A**) HEV induces effective phosphorylation of IRF3: Total cellular extracts from HEV and HEV-UV infected (3 to 72 h p.i.) and poly (I∶C) transfected cells (12 h) were separated on 10% SDS-PAGE and processed for immunoblotting by using anti-IRF3 (Ser396) phospho-specific antibody. The blot was reprobed with IRF3 and anti-actin antibody. (**B**–**C**) Detection of NF-κB p65 subunit in the nuclear and cytoplasmic extracts: A549 cells infected with HEV, HEV-UV and H3N2 or treated with recombinant human TNFα (20 ng/ml) at indicated time points were processed for making nuclear (upper panel) and cytoplasmic (lower panel) extracts, analyzed for immunoblotting with p65 antibody, Respective membrane was stripped and reprobed with anti-nucleolin or anti-actin antibody. Densitometric analysis was performed to quantify the ratio of the density of p65 bands in the nuclear/cytoplasmic fractions to the nucleolin/actin band. The relative density value (RDV) of each band was calculated and normalized RDV values with respect to mock infected sample are represented on the bar graph. An uninfected cell cultures treated with 20 ng/ml of TNFα for 1 and 2 h was included as a positive control to study NF-ĸB activation (Fig. 3C–E). (**D**–**E**) HEV infection induces time dependent IκBα phosphorylation and degradation: Total cellular extracts from HEV infected or TNFα treated cells were analyzed for immunoblotting by using anti-IκBαSer32 phospho specific antibody or anti-IκBα antibody at indicated time points. The densitometric analysis was performed as mentioned in figure 3B–C. (**F**–**G**) UV inactivated HEV is able to induce transient IĸBα phosphorylation and degradation: Total cellular extracts from A549 cells infected with UV inactivated HEV (HEV-UV) at indicated time points were sperated on SDS-PAGE and processed for immunoblotting for detection of phosphorylation followed by degradation of IĸBα as described in figure 3D–E.(**H**) IRF3 or NF-κB -driven firefly luciferase reporter assay: A549 cells were co-transfected with 500 ng of pNF-κB -Luc (firefly luciferase) or pIFN-β-Luc and 5 ng of pGL4.75 hRluc/CMV (Renilla luciferase). Transfected cells were infected after 24 h with either HEV or UV inactivated HEV. Cell lysates were assayed for dual luciferase activity. Results are given as Log_2_ values of relative light units obtained from three independent experiments.

As an immediate response to virus infection, induced innate immune pathways typically converge and activate transcription factors such as NF-κB and IRF3. Activated factors are then transported into the nucleus where they induce IFN and inflammatory cytokine genes. Normally, IRF3 stays in the cytoplasm as an inactive protein but upon phosphorylation forms complex with CREB binding protein (CBP) and gets translocated to the nucleus where it induces IFN gene transcription. Analysis of total cell lysate from HEV infected cells showed increased intensity of the phosphorylated IRF3 protein band from 3–72 h post HEV infection. Phosphorylation of IRF3 remained at the basal level in HEV-UV infected cells (Figure 3A).

NF-κB is a heterodimeric (p65/p50) transcription factor, normally sequestered within the cytoplasm as a latent complex by IkBα. Stimulus-induced phosphorylation of p65 subunit of NF-κB at Ser536 followed by nuclear translocation is critical for DNA binding of NF-κB p65/p50. In HEV infected cells, nuclear accumulation of p65 was evident from 24–72 h p.i. (Figure 3B, upper panel) which was corroborated well with the decrease in the cytoplasmic p65 at respective time points (Figure 3B lower panel). As shown in Fig. 3C, level of nuclear p65 remained same in both mock infected and cells infected with UV inactivated virus. Treatment of cells with TNFα (20 ng/ml) showed increased translocation of p65 at 1 h and 2 h. HEV induced IĸBαSer32 phosphorylation followed by its simultaneous; time dependent degradation (Figure 3D–E) confirmed the involvement of NF-κB pathway in HEV infection. Phosphorylation of IκBα was barely detectable in the cells infected with UV inactivated virus at 12 h (Figure 3F) though minimal level of IκBα degradation was evident at 24 and 48 h. p.i. as compared to mock infected cells (Figure 3G) Taken together, this data suggested activation of both IRF3 and NF-κB transcription factors in HEV infected cells.

### IFN-β and NF-κB promoter activities

As detected by the luciferase reporter assays IFN-β promoter though initially (12–24 h) inactive showed activation (14–16 folds) from 48–96 h p.i. (Figure 3H). However, IFN-β promoter remained completely inactive with the HEV-UV (data not shown).

The NF-κB promoter activation was observed 12 h onwards in both live and HEV-UV infected cells (17–20 fold induction) (Figure 3H).

### Signaling adaptors

**Figure 4:**
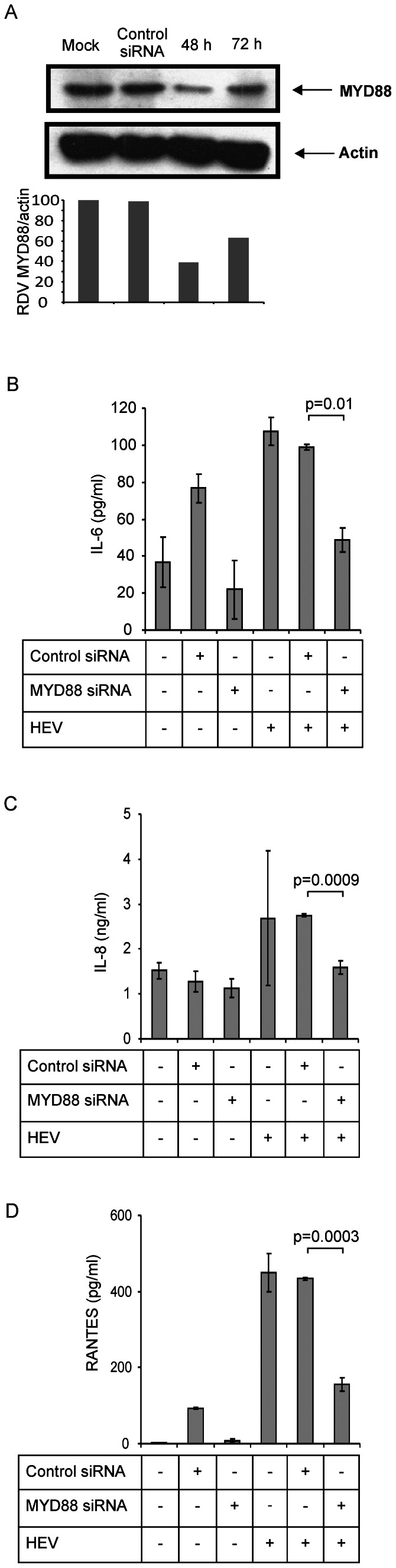
Hepatitis E virus induced secretion of inflammatory cytokines/chemokines crucially requires MyD88 adaptor. A549 cells were transfected either with non-target control siRNA or MyD88 siRNA and infected with HEV and monitored for secreted cytokines/chemokines. (**A**) MyD88 protein levels: Cell lysates of mock transfected, control siRNA transfected and MyD88 siRNA transfected cells were analyzed on 10% SDS-PAGE and the blots were stained using anti-MyD88 and anti-actin antibodies. The densitometric ratio of each time point was compared relative to the steady state ratio of mock cells (lane 1) which was set at 100% and percent (%) reduction of the protein after siRNA knock down was calculated. (**B**–**D**) Knockdown of MyD88 adaptor protein reduced HEV induced secretion of (B) IL-6, (C) IL-8 and (D) RANTES: IL-6, IL-8 and RANTES levels in the culture supernatants were assessed by ELISAs, 24 h post-infection. Culture supernatants from the unifected cells and transfected cells with control non-target siRNA were similarly analysed for ELISAs and served as controls also for the experiments given in figure 5 and 6. The data represents mean ± SD of three independent experiments.

**Figure 5:**
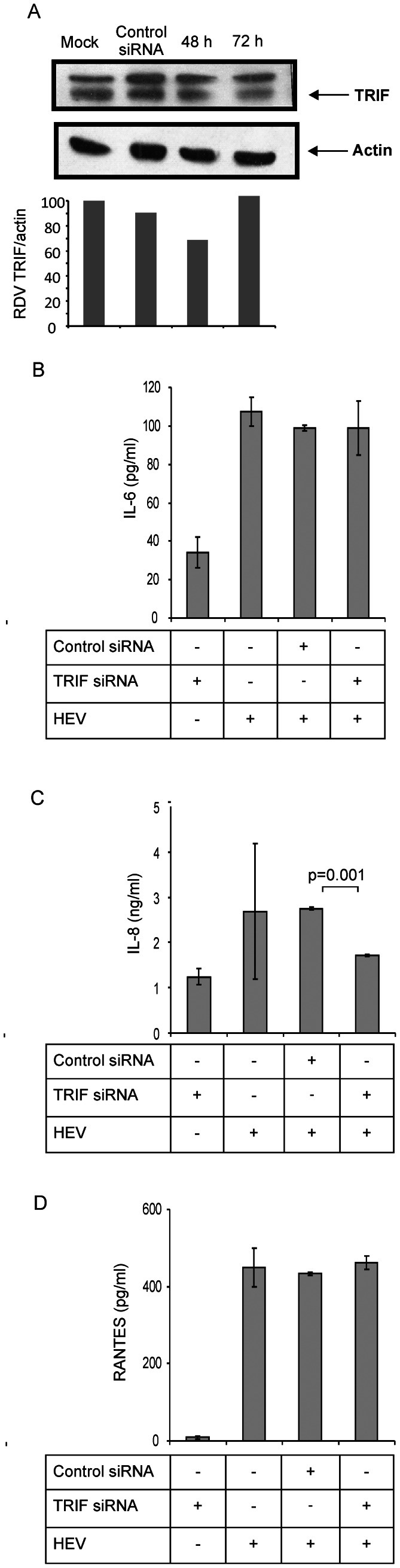
TRIF adaptor knockdown reduces IL-8 induction in HEV infected cells. A549 cells were transfected either with non-target control siRNA or TRIF siRNA, infected with HEV and monitored for secreted cytokines/chemokines. (**A**) A549 cells were analyzed by immunoblotting for TRIF protein levels (arrow indicates specific band for TRIF) as explained for figure 4A. Actin was used as a loading control. Densitometric analysis was performed as described in Figure 4A. (**B**–**D**) IL-6 (B), IL-8 (C) and RANTES (D) levels in the culture supernatants were assessed by ELISA, 24 h post-infection. The data represents mean ± SD of three independent experiments.

**Figure 6:**
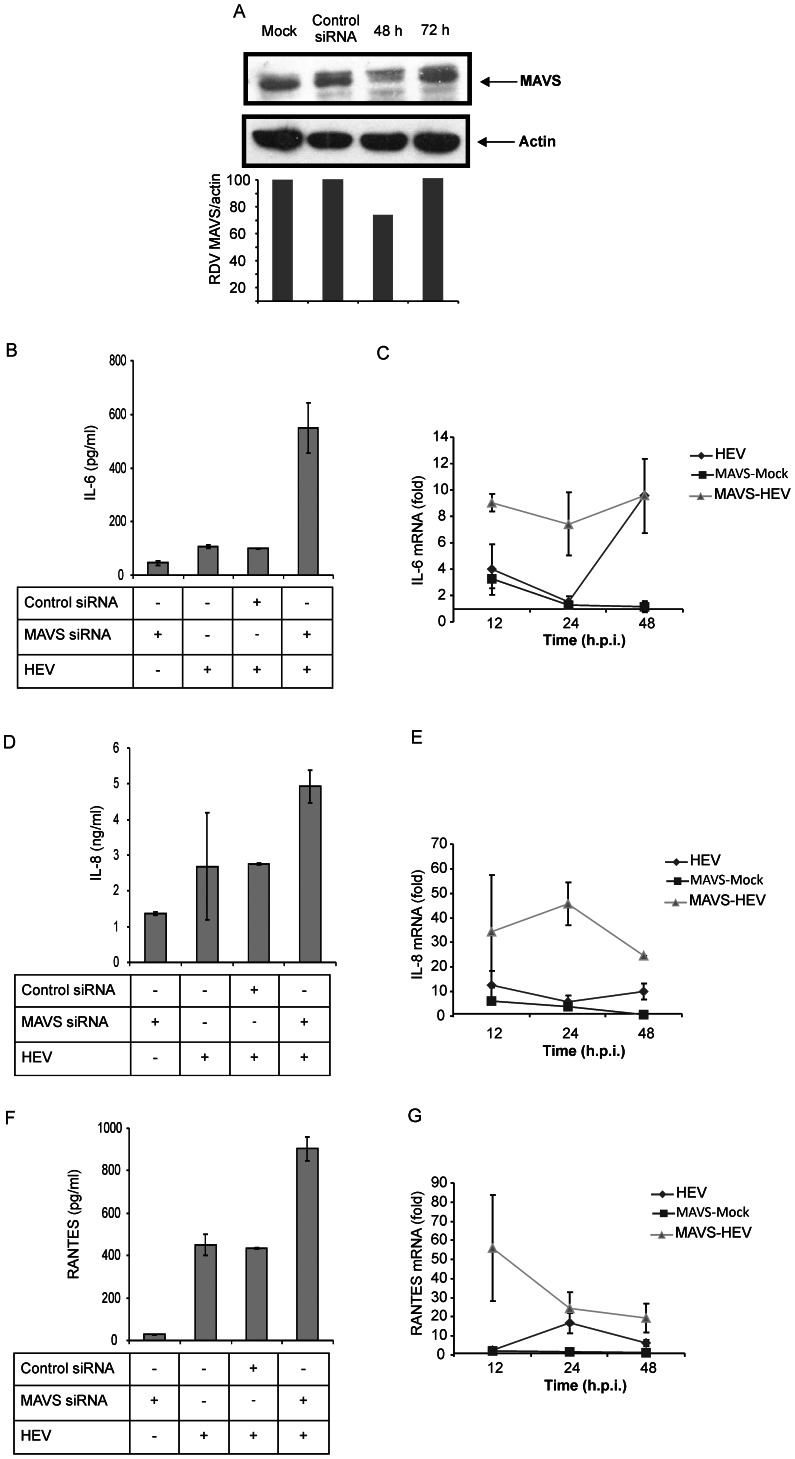
MAVS is dispensable for HEV elicited inflammatory response. (**A**) A549 cells transfected with control non-target siRNA or MAVS siRNA were analyzed by immunoblotting for MAVS protein levels as mentioned in figure 4A. Actin was used as a loading control. Densitometric analysis was performed as in Figure 4A. (**B**–**G**) IL-6 (B, C), IL-8 (D, E) and RANTES (F, G) levels in the culture supernatants were assessed by respective ELISAs and the corresponding cytokine mRNA levels were analyzed by quantitative real time PCR and normalized to that of GAPDH at 24 h post-infection. The data represents mean ± SD of three independent experiments.

TLR signaling is roughly divided into two distinct pathways based on the usage of either MyD88 or TRIF adaptor molecules. On the other hand, RLRs interact with the N-terminal caspase activation and recruitment domain-containing adaptor: MAVS. To identify the adaptors responsible for inducing antiviral response in HEV infected A549 cells, we used siRNAs to target TRIF, MyD88 and MAVS encoding mRNAs. Cell lysates were analyzed by Western blot. As shown in the Figures 4A, 5A, and 6A, adaptor protein levels were significantly reduced (60%, 35% and 30% respectively) at 48 h when compared to mock transfected cells indicating successful reduction of respective proteins.

Cells with MyD88 suppression showed significant reduction (50%) in the levels of secreted IL-6 in HEV infected cells at 24 h when compared to control siRNA (p = 0.01) (Figure 4B). There was no effect of TRIF knockdown on IL-6 levels. This clearly indicated involvement of TLR2 and/or TLR4 in recognizing the viral capsids as a pathogen signal and inducing key inflammatory cytokine IL-6.

IL-8 secretion was affected (40% and 38% respectively) by both MyD88 and TRIF knockdown in HEV infected cells at 24 h (p = 0.0009 and 0.0013 respectively) (Figure 4C, 5C) and the inhibition continued (60% and 30% respectively) till 48 h p.i. (data not shown). Contribution of both MyD88 and TRIF pointed towards involvement of TLR2/TLR4 and TLR3 in recognizing viral capsid and viral dsRNA respectively.

RANTES induction was down-regulated only by MyD88 knockdown (p = 0.0003) at 24 h post HEV infection (Figure 4D). This indicated ability of HEV capsid alone in triggering the response. Taken together, these results show MyD88 and TRIF mediated induction of inflammatory proteins in HEV infected A549 cells.

MAVS knockdown resulted in significant up-regulation of IL-6, IL-8 and RANTES secretions by the HEV infected cells. The respective transcript levels were also significantly up-regulated than the controls confirming the ELISA results (Figure 6B–G). However, there was no increased activation of the promoters (data not shown) indicating involvement of either different transcription regulatory factors or altogether different regulatory mechanism.

We used influenza virus as a control to confirm the expected effect of MyD88, TRIF and MAVS knockdown as this virus induces pathways involving all three adaptors while triggering antiviral response [31]. IL-6 induction in influenza virus infection was inhibited (40%) at 24 h time point in MyD88 suppressed cells while RANTES induction was inhibited by both TRIF and MyD88 suppressed cells (Fig. S3A and C). As expected, influenza virus triggered robust antiviral response with involvement of all the three adaptors.

## Discussion

The innate immune system is the major contributor of acute inflammation induced by microbial infections [3]. Though macrophages and DCs play important roles in development of this response, nonprofessional cells such as epithelial cells, endothelial cells, and fibroblasts are also involved in this [32]. It is not yet known how HEV overcomes the effects of host cellular immunity during the initial phases of establishment in the host cells. Work presented in this manuscript shows analysis of antiviral responses in A549 cells after HEV infection.

Significant up-regulations of inflammatory chemokine genes, RANTES and CCL20 were seen in the HEV infected cells (Table 1 and Fig. S1). CCL20 has been shown to mediate recruitment of CCR6 (CCL20 receptor) expressing leukocytes early upon infections. CCR6 is present on the surface of immature DCs, B and subsets of T cells including effector/memory T cells, Th17 and T regulatory cells. Several studies have identified CCL20/CCR6 interactions contributing to the pathology of inflammatory conditions as well as amplifying local immune response in inflamed liver [33]. RANTES was also consistently up-regulated at both transcription and translation levels in HEV and HEV-UV infected cells. RANTES is known to be secreted by fibroblasts and epithelial cells in viral infections resulting in enhanced leukocyte recruitment. Intra-hepatic expression of RANTES has been positively correlated with the severity of hepatic inflammation in chronic hepatitis C [34]. In view of this, both CCL20 and RANTES require more in depth studies to understand their roles in HEV induced liver inflammation.

Comparable secretion of IL-6, IL-8 and TNF-α by cells infected with HEV and HEV-UV suggested triggering by HEV capsid. Furthermore, inability of baculovirus expressed soluble capsid protein to elicit inflammatory response suggested recognition of viral capsid structure and not soluble capsid protein molecules by the cells. Significant up-regulation of inflammatory genes selectively in live HEV infected cells as compared to HEV-UV indicated involvement of newly synthesized viral molecules in enhancing initial signal by the capsid. For viruses such as influenza, HIV-1, HTLV-1 and HBV it is known that even a short interaction between viral and cellular surface proteins can trigger cellular response leading to the first wave of cytokine production [35]. HEV does not induce apoptosis in infected cells and it is proposed that liver damage due to HEV infection is immune mediated. Prabhu et al., [36] have shown adequate presence of CD8+T cells in the liver biopsies of HEV infected patients suggesting their major role in HEV pathogenesis. Robust inflammatory response initiated at the early stages of HEV infection, as seen in our results, has the potential to initiate massive infiltration of lymphocytes in the liver and in turn result into immune mediated damage to the tissue.

Up-regulated levels of TLR2 and TLR4 and induction of similar inflammatory response with HEV and HEV-UV led us to speculate involvement of these cell surface TLRs in recognizing the viral capsid. It is documented that TLR2 and TLR4 recognize viral capsid proteins and envelope glycoproteins in measles virus, hepatitis C virus, murine leukemia virus, mouse mammary tumor virus and coxsackievirus B4 virus infections [4]. Parallel up-regulation of IRAK2 transcript levels in HEV infected cells supported our speculation of TLR involvement as IRAK2 has been shown to interact with TRAF6 and MyD88 resulting in activation of NF-κB [37]. NF-kB responsive A20/TNFAIP3 gene is known to down-regulate NF-kB signaling through the cooperative activity of its two ubiquitin-editing domains [38]. A significant up-regulation of A20 transcripts in HEV infected cells during 12–96 h period suggested autoregulation of NF-kB levels (Table 1) and attempt of the host cells to keep check on the inflammatory signal.

The low level expression of IFN-α, β and ω genes at 12–24 h correlated well with the initial basal level expression and later (48 h) lower level up-regulation of ISGs (Table S2). This was expected as there were no secreted IFNs in the cell supernatants to induce ISGs in the neighboring, uninfected cells. However, in the infected cells, the lower level up-regulation of type I IFNs possibly resulted in up-regulation of ISGs in autocrine manner. There was no induction of IFNs in HEV-UV infected cells. There is recent report documenting ability of HEV in downregulating IFN-α signaling in A549 cells via ORF3 mediated inhibition of STAT1 phosphorylation [18]. Due to absence of secreted IFNs in our experiments, it is difficult to comment upon the role of ORF3 in keeping lower levels of ISGs in HEV infected cells.

All TLRs except TLR3, initiate signaling through MyD88 adaptor while TLR3 recognising viral dsRNA recruits TRIF which is also shared by TLR4 [5]. RIG-I and MDA5 interact with MAVS and trigger signaling pathways which are also activated by TLRs [39]. Our siRNA experiments convincingly showed dependence of IL-6 and RANTES expression on MyD88 pathway in HEV infected cells. This suggested involvement of TLR2 and/or TLR4 in sensing HEV capsids (Figure 4). IL-8 expression was reduced by both MyD88 and TRIF siRNAs (∼40% in both cases) (Figure 4B, 5C). This indicated possible involvement of TLR3 and TLR4 in sensing HEV.

MAVS knockdown surprisingly resulted in increased secretion of IL-6, IL-8 and RANTES by HEV infected cells, without increasing the IFN secretion. MAVS being the sole adaptor for all RLRs, these results indicated absence of RLR trigger in eliciting antiviral response against HEV in A549 cells. On the contrary, it indicated that HEV recruits MAVS/MAVS mediated pathways in keeping check on the inflammatory response.

HEV replicates its genome via dsRNA intermediates which are expected to be sensed either by RLRs (RIG-I/MDA5) or by TLR3. Our results indicated that by some means HEV manages to restrict RLR mediated innate immune response. This could be due to, i) HEV replication occurs in restricted compartments within the cells where viral RNA remains undetected by the cytosolic sensors or ii) HEV actively downregulates MAVS mediated induction of inflammatory response. There is indirect evidence that HEV replicates in the endoplasmic reticulum [40]. With our observations showing involvement of TRIF in addition to MyD88 in inducing inflammatory response it can be hypothesized that, dsRNA synthesized during HEV replication is recognized by TLR3 present in the endosomal compartments leading to TRIF mediated IFN activation. Complete absence of IFNs with HEV-UV infection supports this presumption. We have recently documented association of deubiquitination and deISGylation activity with the papain like cysteine protease (PCP) domain in HEV ORF1 [41]. N-terminal caspase recruitment domain of RIG-I is known to undergo robust ubiquitination and activation, initiating antiviral response in mammalian cells [42]. It would be interesting to see whether HEV PCP is involved in any way in downregulating RLR mediated signaling.

In conclusion, HEV induces MyD88 and TRIF mediated activation of IRF3 and NF-κB, apparently via TLR2, TLR3 and TLR4 sensing, leading to inflammatory response. It is now an established fact that the innate response greatly influences subsequent adaptive response in viral infections. Use of lung epithelial cells, A549, which are not natural target cells for HEV infection, is the main limitation of this study. Immune cell activation is differentially regulated depending on the cell type involved and it would be worthwhile to see the response of different cell types such as primary hepatocytes, Kupffer cells (liver macrophages), macrophages and dendritic cells to HEV infection. It is known that HEV induced fulminant hepatic failure occurs due to immune dysfunction. This study has generated several unanswered questions which warrant further work to understand HEV pathogenesis.

## Supporting Information

Figure S1 **Relative gene expression in infected cells.** A549 cells were infected either with HEV, UV inactivated HEV or H3N2 virus, total RNA was extracted and processed for quantitative real-time PCR to detect mRNAs for (A) IFN-α (B) IFN-β, (C) IL-6, (D) RANTES, (E) IL-8 and (F) TNF-α. The results were normalized by GAPDH expression and are presented as relative up- or down-regulation in comparison with mock-infected cells. Results correspond to mean ± SD of RQ (Relative quantitation) values obtained from three separate experiments.
(TIF)

Figure S2 **Influenza A infection induces production of inflammatory cytokines/chemokines in A549 cells.** Cell culture supernatants from A549 cells infected with H3N2 virus were tested for IL-6 (A), IL-8 (B), RANTES (C) and TNFα (D) by ELISA. Data are mean ± SD of four independent experiments.
(TIF)

Figure S3 **Influenza A virus infection elicits inflammatory response by recruiting TLR and RLR adaptors.** (A–C) A549 cells transfected with non target control siRNA or MyD88, TRIF and MAVS siRNAs were infected with H3N2 virus (MOI = 1) and the accumulation of IL-6 (A), IL-8 (B) and RANTES (C) in the culture supernatants was assessed by ELISA 24 h post-infection. Data presented are mean ± SD of two independent experiments.
(TIF)

Table S1 **List of the genes assayed by TaqMan Low Density Array (TLDA).**
(DOCX)

Table S2 **Primer sequences used for real-time PCR assays.**
(DOCX)

Table S3 **Gene expression analysis of A549 cells infected with HEV, UV inactivated HEV and H3N2 virus.**
(DOCX)
