# Supplementary material for: Analysis of Antiviral Response in Human Epithelial Cells Infected with Hepatitis E Virus
Source: PLoS One. 2013 May 9;8(5):e63793. doi: 10.1371/journal.pone.0063793 (PMC3650073; doi:10.1371/journal.pone.0063793)
Supplement: Table S1 — List of the genes assayed by TaqMan Low Density Array (TLDA). (DOCX) [file pone.0063793.s004.docx]

**Table S1.** **List of the genes assayed by TaqMan Low Density Array (TLDA)**

| Detector | ABI Assay ID | Description |
| --- | --- | --- |
| 18s rRNA | Hs99999901_s1 | Eukaryotic 18s rRNA |
| ADAR | Hs00241666_m1 | adenosine deaminase, RNA-specific |
| ATF1 | Hs00909673_m1 | activating transcription factor 1 |
| B2M | Hs00187842_m1 | beta-2-microglobulin |
| CASP8 | Hs01018151_m1 | caspase 8, apoptosis-related cysteine peptidase |
| CCL20 | Hs00355476_m1 | chemokine (C-C motif) ligand 20 |
| CD40 | Hs99999100_s1 | CD40 molecule, TNF receptor superfamily member 5 |
| CFLAR | Hs01116280_m1 | CASP8 and FADD-like apoptosis regulator |
| CXCL10 | Hs00171042_m1 | chemokine (C-X-C motif) ligand 10 |
| CXCL9 | Hs00171065_m1 | chemokine (C-X-C motif) ligand 9 |
| DDX58 | Hs00204833_m1 | DEAD (Asp-Glu-Ala-Asp) box polypeptide 58 |
| DHX58 | Hs00225561_m1 | DEXH (Asp-Glu-X-His) box polypeptide 58 |
| EIF2AK2 | Hs00169345_m1 | eukaryotic translation initiation factor 2-alpha kinase 2 |
| FADD | Hs00538709_m1 | Fas (TNFRSF6)-associated via death domain |
| FAS | Hs99999006_m1 | Fas (TNF receptor superfamily, member 6) |
| GBP1 | Hs00266717_m1 | guanylate binding protein 1, interferon-inducible, 67kDa |
| GBP2 | Hs00894837_m1 | guanylate binding protein 2, interferon-inducible |
| HPRT1 | Hs02800695_m1 | hypoxanthine phosphoribosyltransferase 1 |
| IFI27 | Hs00271467_m1 | interferon, alpha-inducible protein 27 |
| IFI44 | Hs00197427_m1 | interferon-induced protein 44 |
| IFIH1 | Hs00223420_m1 | interferon induced with helicase C domain 1 |
| IFIT1 | Hs01911452_s1 | interferon-induced protein with tetratricopeptide repeats 1 |
| IFIT2 | Hs00533665_m1 | interferon-induced protein with tetratricopeptide repeats 2 |
| IFNA1 | Hs00256882_s1 | interferon, alpha 1 |
| IFNAR1 | Hs00265057_m1 | interferon (alpha, beta and omega) receptor 1 |
| IFNAR2 | Hs00174198_m1 | interferon (alpha, beta and omega) receptor 2 |
| IFNB1 | Hs00277188_s1 | interferon, beta 1, fibroblast |
| IFNG | Hs00174143_m1 | interferon, gamma |
| IFNGR2 | Hs00194264_m1 | interferon gamma receptor 2 (interferon gamma transducer 1) |
| IFNW1 | Hs00357857_s1 | interferon, omega 1 |
| IKBKB | Hs00826074_m1 | inhibitor of kappa light polypeptide gene enhancer in B-cells, kinase beta |
| IKBKE | Hs00203911_m1 | inhibitor of kappa light polypeptide gene enhancer in B-cells, kinase epsilon |
| IKBKG | Hs00175318_m1 | inhibitor of kappa light polypeptide gene enhancer in B-cells, kinase gamma |
| IL10 | Hs00174086_m1 | interleukin 10 |
| IL18 | Hs99999040_m1 | interleukin 18 (interferon-gamma-inducing factor) |
| IL6 | Hs00174131_m1 | interleukin 6 (interferon, beta 2) |
| IL8 | Hs99999034_m1 | interleukin 8 |
| IRAK1 | Hs00155570_m1 | interleukin-1 receptor-associated kinase 1 |
| IRAK2 | Hs00176394_m1 | interleukin-1 receptor-associated kinase 2 |
| IRF1 | Hs00971960_m1 | interferon regulatory factor 1 |
| IRF3 | Hs00155574_m1 | interferon regulatory factor 3 |
| IRF7 | Hs00185375_m1 | interferon regulatory factor 7 |
| IRF9 | Hs00196051_m1 | interferon regulatory factor 9 |
| ISG15 | Hs01921425_s1 | ISG15 ubiquitin-like modifier |
| JAK1 | Hs01026983_m1 | Janus kinase 1 |
| JAK2 | Hs00234567_m1 | Janus kinase 2 |
| JAK3 | Hs00169663_m1 | Janus kinase 3 |
| LTA | Hs00236874_m1 | lymphotoxin alpha (TNF superfamily, member 1) |
| MAVS | Hs00393845_m1 | mitochondrial antiviral signaling protein |
| MX1 | Hs00895608_m1 | myxovirus (influenza virus) resistance 1, interferon-inducible protein p78 |
| MX2 | Hs01550808_m1 | myxovirus (influenza virus) resistance 2 |
| MYD88 | Hs00182082_m1 | myeloid differentiation primary response gene (88) |
| NFKB1 | Hs00765730_m1 | nuclear factor of kappa light polypeptide gene enhancer in B-cells 1 |
| NFKB2 | Hs00174517_m1 | nuclear factor of kappa light polypeptide gene enhancer in B-cells 2 (p49/p100) |
| NFKBIB | Hs00182115_m1 | nuclear factor of kappa light polypeptide gene enhancer in B-cells inhibitor, beta |
| NOS2 | Hs01075529_m1 | nitric oxide synthase 2, inducible |
| OAS1 | Hs00242943_m1 | 2,5-oligoadenylate synthetase 1 |
| OAS2 | Hs00942643_m1 | 2-5-oligoadenylate synthetase 2, 69/71kDa |
| PIAS1 | Hs00184008_m1 | protein inhibitor of activated STAT, 1 |
| PIAS2 | Hs00190699_m1 | protein inhibitor of activated STAT, 2 |
| PIK3CB | Hs00927728_m1 | phosphoinositide-3-kinase, catalytic, beta polypeptide |
| PRKRA | Hs00269379_m1 | protein kinase, interferon-inducible double stranded RNA dependent activator |
| PSME2 | Hs01923165_u1 | proteasome (prosome, macropain) activator subunit 2 (PA28 beta) |
| RSAD2 | Hs01057264_m1 | radical S-adenosyl methionine domain containing 2 |
| RIPK1 | Hs00169407_m1 | receptor (TNFRSF)-interacting serine-threonine kinase 1 |
| SOCS1 | Hs00705164_s1 | suppressor of cytokine signaling 1 |
| SOCS2 | Hs00919620_m1 | suppressor of cytokine signaling 2 |
| SOCS3 | Hs02330328_s1 | suppressor of cytokine signaling 3 |
| SOCS4 | Hs00328404_s1 | suppressor of cytokine signaling 4 |
| SOCS5 | Hs00367107_m1 | suppressor of cytokine signaling 5 |
| STAT1 | Hs00234829_m1 | signal transducer and activator of transcription 1, 91kDa |
| STAT2 | Hs00237139_m1 | signal transducer and activator of transcription 2, 113kDa |
| STAT3 | Hs00374280_m1 | signal transducer and activator of transcription 3 (acute-phase response factor) |
| STAT4 | Hs01028017_m1 | signal transducer and activator of transcription 4 |
| TANK | Hs00370305_m1 | TRAF family member-associated NFKB activator |
| TAP1 | Hs00388682_m1 | transporter 1, ATP-binding cassette, sub-family B (MDR/TAP) |
| TAPBP | Hs00175269_m1 | TAP binding protein (tapasin) |
| TBK1 | Hs00179410_m1 | TANK-binding kinase 1 |
| TICAM1 | Hs00706140_s1 | toll-like receptor adaptor molecule 1 |
| TIRAP | Hs00364644_m1 | toll-interleukin 1 receptor (TIR) domain containing adaptor protein |
| TLR3 | Hs00152933_m1 | toll-like receptor 3 |
| TLR7 | Hs00152971_m1 | toll-like receptor 7 |
| TLR8 | Hs00152972_m1 | toll-like receptor 8 |
| TNF | Hs00174128_m1 | tumor necrosis factor (TNF superfamily, member 2) |
| TNFAIP3 | Hs00234713_m1 | tumor necrosis factor, alpha-induced protein 3 |
| TRAF3 | Hs00377462_m1 | TNF receptor-associated factor 3 |
| TRAF6 | Hs00270336_m1 | TNF receptor-associated factor 6 |
| TNFSF10 | Hs00921974_m1 | tumor necrosis factor (ligand) superfamily, member 10 |
| XIAP | Hs00236913_m1 | X-linked inhibitor of apoptosis |
| XAF1 | Hs00213882_m1 | XIAP associated factor 1 |
| TLR2 | Hs00152932_m1 | toll-like receptor 2 |
| TLR4 | Hs00370853_m1 | toll-like receptor 4 |
| IL12A | Hs99999036_m1 | interleukin 12A (natural killer cell stimulatory factor 1, cytotoxic lymphocyte maturation factor 1, p35) |
| TNFSF10 | Hs00234356_m1 | tumor necrosis factor (ligand) superfamily, member 10 |
| TRIM25 | Hs01116121_m1 | tripartite motif-containing 25 |
| ATF5 | Hs00247172_m1 | activating transcription factor 5 |
