# Supplementary material for: Analysis of Antiviral Response in Human Epithelial Cells Infected with Hepatitis E Virus
Source: PLoS One. 2013 May 9;8(5):e63793. doi: 10.1371/journal.pone.0063793 (PMC3650073; doi:10.1371/journal.pone.0063793)
Supplement: Table S2 — Primer sequences used for real-time PCR assays. (DOCX) [file pone.0063793.s005.docx]

**Table S2.** **Primer sequences used for real-time PCR assays.**

| Primer | Orientation | Sequence | Product size (bp) |
| --- | --- | --- | --- |
| TNF-α | Sense | CAGAGGGAAGAGTTCCCCAGGGACC | 325 |
|  | Antisense | CCTTGGTCTGGTAGGAGACGG |  |
| IL-6 | Sense | TGTGAAAGCAGCAAAGAGGCACTG | 194 |
|  | Antisense | ACAGCTCTGGCTTGTTCCTCACTA |  |
| IL-8 | Sense | CAGCCAAAACTCCACAGTCA | 155 |
|  | Antisense | TTGGAGAGCACATAAAAACATCT |  |
| RANTES | Sense | GCATCTGCCTCCCCATATTC | 64 |
|  | Antisense | CAGTGGGCGGGCAATG |  |
| IFN-α | Sense | GCCTCGCCCTTTGCTTTACT | 88 |
|  | Antisense | CTGTGGGTCTCAGGGAGATCA |  |
| IFN-β | Sense | ATGACCAACAAGTGTCTCCTCC | 68 |
|  | Antisense | GCTCATGGAAAGAGCTGTAGTG |  |
| GAPDH | Sense | GTGGACCTGACCTGCCGTCT | 153 |
|  | Antisense | GGAGGAGTGGGTGTCGCTGT |  |
