# Supplementary material for: Analysis of Antiviral Response in Human Epithelial Cells Infected with Hepatitis E Virus
Source: PLoS One. 2013 May 9;8(5):e63793. doi: 10.1371/journal.pone.0063793 (PMC3650073; doi:10.1371/journal.pone.0063793)
Supplement: Table S3 — Gene expression analysis of A549 cells infected with HEV, UV inactivated HEV and H3N2 virus. (DOCX) [file pone.0063793.s006.docx]

**Table S3.** **Gene expression analysis of A549 cells infected with HEV, UV inactivated HEV and H3N2 virus.**

| Pathway/Function | Gene | HEV and (HEV-UV)* | | | | | H3N2 |
| --- | --- | --- | --- | --- | --- | --- | --- |
|  |  | 12h | 24h | 48h | 72h | 96h | 12h |
| Interferon Receptors | IFNAR1 | 1.5 (0.4) | 1.1(0.7) | 2.4 (0.5) | 1.8 | 1.5 (0.3) | 0.8 |
|  | IFNAR2 | 1.6 (0.7) | 1.5 (0.7) | 2.7 (0.8) | 1.6 | 1.9 (0.4) | 0.3 |
| Interferon Stimulated Genes (ISGs) | EIF2AK2/PKR | 1.2 (0.5) | 1 (0.8) | 3.1 (0.6) | 1.6 | 2 (0.4) | 2.6 |
|  | GBP1 | 1.1 (8.3) | 2.4 (1.4) | 17.6 (2.3) | 1 | 0.6 (1.4) | 16 |
|  | GBP2 | 1.7 (0.3) | 2.3 (1.8) | 5.5 (0.4) | 2.9 | 20 (1.3) | 2.7 |
|  | IFI27 | 0.4 (0.4) | 0.4 (1) | 1.5 (0.6) | 0.9 | 0.6 (0.4) | 110 |
|  | IFI44 | 0.7 (0.5) | 0.9 (3.5) | 5.9 (0.2) | 2.4 | 1.1 (0.1) | 32.8 |
|  | IFIT1 (ISG56) | 0.3 (0.6) | 0.7 (1) | 7 (0.3) | 3.7 | 5.3 (0.7) | 53.5 |
|  | IFIT2 (ISG54) | 0.5 (0.7) | 1.1 (1.9) | 6.2 (0.1) | 7.6 | 43 (1.2) | 118 |
|  | IRF1 | 1.5 (0.9) | 1.7 (1.6) | 3.6 (1.2) | 2 | 2.4 (2) | 4.5 |
|  | IRF7 | 1.1 (0.3) | 0.8 (1.9) | 3 (0.7) | 1.3 | 1.2 (0.3) | 15.8 |
|  | IRF9 | 1.3 (0.1) | 1.5 (1.3) | 6.6 (0.2) | 3.8 | 6.2 (0.5) | 3.1 |
|  | ISG15 | 0.5 (0.4) | 0.6 (1.2) | 2.9 (0.5) | 1.3 | 1.5 (0.9) | 29.5 |
|  | MX1 | 0.5 (0.4) | 0.7 (2) | 24.8 (0.3) | 3.1 | 55.8 (1) | 173 |
|  | OAS1 | 1.4 (0.3) | 1.9 (1.5) | 3.4 (0.4) | 3.3 | 4.3 (0.8) | 14.1 |
|  | RSAD2/Viperin | 0.5 (0.1) | 0.8 (2.5) | 0.6 (0.00) | 1.7 | 1.6 (0.2) | 278 |
|  | B2M | 1.6 (0.6) | 1.7 (1.2) | 4.4 (0.9) | 1.6 | 1.9 (0.5) | 3 |
|  | ADAR | 1 (0.4) | 0.8 (0.7) | 2.8 (0.6) | 1.7 | 2.2 (0.4) | 1.4 |
| IFN signaling | JAK1 | 2 (0.7) | 1.5 (1) | 2.5 (1) | 1.3 | 1.2 (0.4) | 0.9 |
|  | JAK2 | 1.1 (0.1) | 1 (0.8) | 2.6 (0.2) | 3 | 11 (0.6) | 1.9 |
|  | PIAS1 | 1.4 (0.2) | 1.1 (0.9) | 2.4 (0.5) | 1.7 | 1.7 (0.4) | 0.6 |
|  | PIAS2 | 1.1 (0.4) | 1 (1.1) | 2.4 (0.5) | 1.5 | 0.7 (0.2) | 0.6 |
|  | SOCS1 | 0.9 (0.2) | 0.5 (5.6) | 2.5 (0.3) | 1.9 | 19.2 (0.6) | 11 |
|  | SOCS2 | 2 (0.8) | 2.2 (1.4) | 1.1 (0.4) | 0.9 | 1.3 (0.2) | 2.1 |
|  | SOCS3 | 1.7 (0.9) | 2.4 (1.5) | 1.7 (1.2) | 1.2 | 1.3 (0.6) | 3.5 |
|  | STAT1 | 0.7 (0.5) | 1 (1) | 2.2 (1) | 1.6 | 1.3 (0.4) | 6 |
|  | STAT2 | 1.4 (0.3) | 2.1 (1.7) | 3.3 (0.4) | 2.4 | 1.1 (0.3) | 5.6 |
|  | STAT3 | 1.7 (0.1) | 1 (0.9) | 3.6 (0.5) | 2.5 | 4.4 (0.8) | 0.8 |
|  | TRAF3 | 1.7 (0.9) | 1.5 (0.8) | 2.9 (1) | 1.6 | 1.2 (0.5) | 1.038 |
|  | TRAF6 | 1.6 (0.3) | 1.6 (0.9) | 2.7 (0.5) | 1.7 | 1.7 (0.4) | 0.5325 |

Gene expression levels are given as average fold change in expression obtained from three independent experiments compared to mock infected cells, * RQ values obtained from UV inactivated HEV infection.
